# Comparison of volumetric modulated arc therapy and intensity-modulated radiotherapy for left-sided whole-breast irradiation using automated planning

**DOI:** 10.1007/s00066-021-01817-x

**Published:** 2021-08-05

**Authors:** L. Redapi, L. Rossi, L. Marrazzo, J. J. Penninkhof, S. Pallotta, B. Heijmen

**Affiliations:** 1 Department of Experimental and Clinical Biomedical Sciences “Mario Serio”, University of Florence, Florence, Italy; 2 Department of Radiation Oncology, Erasmus MC Cancer Institute, Rotterdam, The Netherlands; 3 Medical Physic Unit, Azienda Ospedaliera Universitaria Careggi, Florence, Italy

**Keywords:** Autoplanning, Breast cancer, Partial arcs volumetric modulated arc therapy, Tangent-based intensity, Modulated radiation therapy, Deep inspiration breath-hold

## Abstract

**Background:**

Published treatment technique comparisons for postoperative left-sided whole breast irradiation (WBI) with deep-inspiration breath-hold (DIBH) are scarce, small, and inconclusive. In this study, fully automated multi-criterial plan optimization, generating a single high-quality, Pareto-optimal plan per patient and treatment technique, was used to compare for a large patient cohort 1) intensity modulated radiotherapy (IMRT) with two tangential fields and 2) volumetric modulated arc therapy (VMAT) with two small tangential subarcs.

**Materials and methods:**

Forty-eight randomly selected patients recently treated with DIBH and 16 × 2.66 Gy were included. The optimizer was configured for the clinical planning protocol. Comparisons between IMRT and VMAT included dosimetric plan parameters, estimated excess relative risks (ERR) for toxicities, delivery times, MUs, and deliverability accuracy at a linac.

**Results:**

The automatically generated IMRT and VMAT plans applied in this study were similar or higher in quality than the manually generated clinical plans. For equal PTVin V95% (98.4 ± 0.9%), VMAT had significant advantages compared to IMRT regarding breast dose homogeneity and doses in heart and ipsilateral lung, at the cost of some minor deteriorations for contralateral breast (few cases with larger deteriorations) and lung. Conformality improved from 1.38 to 1.18 (*p* < 0.001). With VMAT, ERR for major coronary events and ipsilateral lung tumors were reduced by 3% (range: −1–12%) and 16% (range: −3–38%), respectively. MUs and delivery times were higher for VMAT. There were no statistical differences in γ passing rates.

**Conclusion:**

For WBI in conservative therapy of left-sided breast patients treated with DIBH, VMAT with two tangential subarcs was generally dosimetrically superior to IMRT with two tangential static fields. Results need confirmation by robustness analyses.

## Background

Breast cancer is the most commonly occurring cancer in women and the second most common cancer overall, making it one of the main causes of mortality and morbidity in females worldwide [1]. Breast-conserving therapy with limited surgery followed by homogenous irradiation of the whole breast (WBI) is often the procedure of choice for management of early-stage breast cancer [2]. The conventional radiotherapy technique for WBI consists of two opposing tangential fields with wedges. Intensity-modulated radiotherapy (IMRT) with static beams, and later also volumetric arc therapy (VMAT), have been proposed to improve breast dose homogeneity and possibly reduce dose to organs at risk (OAR). IMRT is mostly delivered with two opposing tangential fields with patient-specific intensity-modulated profiles, sometimes combined with two open tangential fields (hybrid approach [3, 4]). For VMAT, the two static tangential IMRT fields are often replaced by two small tangential arcs or by a single, larger partial arc [5, 6].

Improving dose homogeneity in the target and reducing OAR doses in WBI can be clinically advantageous [7–14]. So far, clinical trials comparing IMRT and VMAT for WBI have not been performed. There are few published treatment planning studies for left-sided WBI that compare tangential IMRT with tangential VMAT, all with low numbers of patients [15–19]. Two of these studies [17, 18] included treatments in DIBH. Overall, the literature is inconclusive regarding the choice of IMRT or VMAT for WBI. Apart from the low patient numbers, this may also be related to the applied conventional trial-and-error treatment planning with well-known challenges for consistent high-quality plan generation.

Recently, several systems for automated treatment planning have been proposed for reduced workload and more consistent and higher plan quality [20]. In treatment technique comparisons, planning automation can avoid potential bias caused by human planners and limited planning time [21, 22]. Moreover, due to automation, it is possible to perform comparisons based on large sample sizes [23]. Automated IMRT and VMAT planning have also been investigated for breast cancer radiotherapy [24–33]. A few of these studies applied automated planning for WBI [24–27, 32, 33]. However, in none of them was automated planning used for a systematic comparison between IMRT and VMAT.

The main aim of this study was to systematically compare treatment with either two tangential IMRT fields or with two small tangential VMAT arcs for a large cohort of left-sided DIBH patients, and to use for both treatment approaches fully automatically generated high-quality treatment plans (autoIMRT and autoVMAT). The same optimizer configuration was used for autoIMRT and autoVMAT plan generation, ensuring minimal bias. Dosimetric plan parameters were compared, and parameter differences were used to estimate differences in excess relative risks (ERR) for major coronary events and for ipsilateral lung tumors. Plans were also compared regarding total MU, delivery time, and delivery accuracy as assessed with measurements at a linac. Prior to the autoIMRT/autoVMAT comparisons, autoIMRT and autoVMAT plans were compared to corresponding manually generated, clinically delivered plans (clinical), in order to ensure clinical relevance of the former plans.

## Materials and methods

### Patients

Forty-eight randomly selected left-sided breast cancer patients recently treated in Erasmus MC with postoperative WBI without a boost dose were included. Patients were treated with DIBH using daily CBCT setup correction and intrafractional monitoring with an optical system (Vision RT, London, UK). Planning CTs were acquired in DIBH with 3 mm slice thickness. The clinical target volume (CTV), heart, ipsilateral lung, contralateral lung, and contralateral breast were manually contoured. A planning target volume (PTV) was generated with 5 mm margin to the CTV and then cropped 5 mm from the skin surface, referred to as PTVin in the remainder of the paper. Patients needing a high skin dose were not included in our study. As described below, a flash margin was used to enhance robustness of CTV dose delivery. The average PTVin volume of the study cohort was 865 ± 310 cc (range 337–1892 cc).

### Clinical planning goals

A hybrid-IMRT technique was used to deliver 42.56 Gy in 16 fractions. The planning was performed according to ALARA principles for OARs with an accent on the following goals: PTVin V_95%_ ≤ 95%, PTVin Dmax ≤ 107% (prescribed dose = 100%), mean dose in ipsilateral lung < 10 Gy and heart < 3 Gy. Tangential beams were chosen such that the projected area of the PTVin in the beams-eye-view was small, overlap between PTVin and heart and ipsilateral lung was minimal, and overlap with the contralateral breast was avoided. Dose < 95% of the prescribed dose in small volumes at the mediodorsal part of the PTVin was accepted if heart dose could be lowered without compromising CTV coverage.

### Automated treatment planning—system and configuration

The generation of autoIMRT and autoVMAT plans was performed with Erasmus-iCycle, a system for fully automated multi-criterial fluence map optimization (FMO) [34, 35], which was coupled to the Monaco TPS (Elekta AB, Stockholm, Sweden) for automatic conversion of FMO plans into deliverable (segmented) plans. The system generates a single Pareto-optimal IMRT or VMAT plan for each patient fully automatically. As confirmed in validation studies for many anatomical sites [35–40], with proper system configuration, the generated plans are of high clinical quality. For each anatomical site, system configuration entails creation of a specific optimization protocol called wish-list, containing objective functions with assigned priorities and goal values, and hard constraints that are never violated. For this study, a single wish-list was constructed for automated generation of all autoIMRT and all autoVMAT plans, which was in line with the clinical planning aims. Manual fine-tuning of automatically generated plans was not performed.

### Treatment plans

All final plans were generated with Monaco (version 5.11.02, Elekta AB, Stockholm, Sweden) using the Monte Carlo dose calculation algorithm. In all plans, the clinical isocenter selected by the clinical planner was used.

- *Clinical*: The manually generated hybrid-IMRT plans delivered about 70% of the prescription dose with two tangential open fields covering the total breast, and 30% with two sliding-window IMRT beams, inclined by 5º from the open beams towards the left-right axis. The beam energy was 6 MV, 10 MV, or a combination, depending on breast size.
- *Automated: *For each patient, the beam angles for the two tangential IMRT fields in the autoIMRT plan were the same as used in the clinical plan. For autoVMAT, two partial dual arcs were used with fixed arc lengths of 60° and fixed start and return angles (100°–160° and 290°–350°). The plans were generated for an Elekta linac with an Agility MLC and a fixed beam energy of 6 MV. In the applied step-and-shoot IMRT, the allowed minimum segment area was 3 cm^2^, while the minimum segment width was set to 1 cm, and the minimum number of MU per segment was set to 4. For VMAT, limiting the minimum segment area is not possible, but the minimum segment width was set to 1 cm. In order to avoid large modulation, the fluence-smoothing parameter in the Monaco TPS was set to medium. For both techniques, a skin flash margin of 2 cm was applied to extend fluence outside the body contour to make the plan more robust for anatomical variations, such as breast swelling. In the auto-flash margin option of the Monaco TPS, the amount of the skin flash can be defined by the user up to 2.5 cm, and the leaves are extended only at gantry angles where they would be otherwise limited to the skin. In the auto-flash margin option no additional optimization bolus or body contour extensions are needed in order to obtain the aperture of segments extending outside the patient.

### Dosimetric plan evaluation and comparison

Prior to the dosimetric analyses, for each patient, the autoIMRT and autoVMAT plans were normalized to have the same PTVin coverage (V95%) as the corresponding clinical plan. The following plan parameters were then considered: PTVin: D98%, V105%; ipsilateral lung: Dmean, V5Gy, and V20Gy; heart: Dmean and near-maximum dose (D1cc); contralateral lung: Dmean and V5Gy; and contralateral breast: Dmean and V5Gy. The conformity index (CI), defined as the ratio between the 95% isodose volume and the PTVin volume, was used to assess plan conformity. Dose homogeneity in the PTVin was quantified using the homogeneity index (HI), defined as the difference between D2% and D98% divided by the prescription dose. Finally, the low-dose bath was evaluated with V2Gy, and integral dose (ID, defined as the mean dose times volume) to the patient structure without PTVin (patient-PTVin).

### Risks for radiation-induced side effects

Several studies have observed enhanced risks for cardiac toxicity and secondary lung cancer after breast cancer radiotherapy [11, 12, 41, 42]. In this study, we estimated differences between autoIMRT and autoVMAT in excess relative risks (ERR = ((radiation induced)/expected)*100%) for major coronary events and ipsilateral lung cancer based on differences in dosimetric plan parameters and published dependences of ERR on radiotherapy dose. For major coronary events, an ERR of 7.4% for every Gy increase in heart Dmean was assumed, following Darby et al. [41]. ERR for ipsilateral lung tumors were estimated assuming ERR increases of 8.5% per Gy mean lung dose [12].

### Plan deliverability, monitor units, and delivery times

To investigate deliverability of autoIMRT and autoVMAT plans, dosimetric verification measurements were performed for 10 arbitrarily selected patients using an Octavius phantom with a 2D-array729 (PTW Freiburg GmbH). The Erasmus MC QA protocol was used to assess plan acceptability: plans were considered clinically deliverable when the γ pass rate (global approach; 3%; 3 mm) was > 90% and the mean γ was < 0.5. Measurements at gantry angle zero were prescribed when γ pass rates were lower, in order to rule out angular dependency of the detector array. For the 10 selected patients, delivery times and MU were measured during QA.

### Statistical analyses

Statistical significance of plan parameter differences between planning strategies was evaluated using paired two-sided Wilcoxon’s signed-rank tests with a significance level of 0.05.

## Results

Normalization of autoIMRT and autoVMAT plans to obtain the same PTVin coverage as the corresponding clinical plans resulted in an average PTVin V95% of 98.4 ± 0.9%, range 94.9–99.7. Rescaling factors were small: 0.995 ± 0.010 for both techniques.

### Clinical vs. autoplanning

**Fig. 1:**
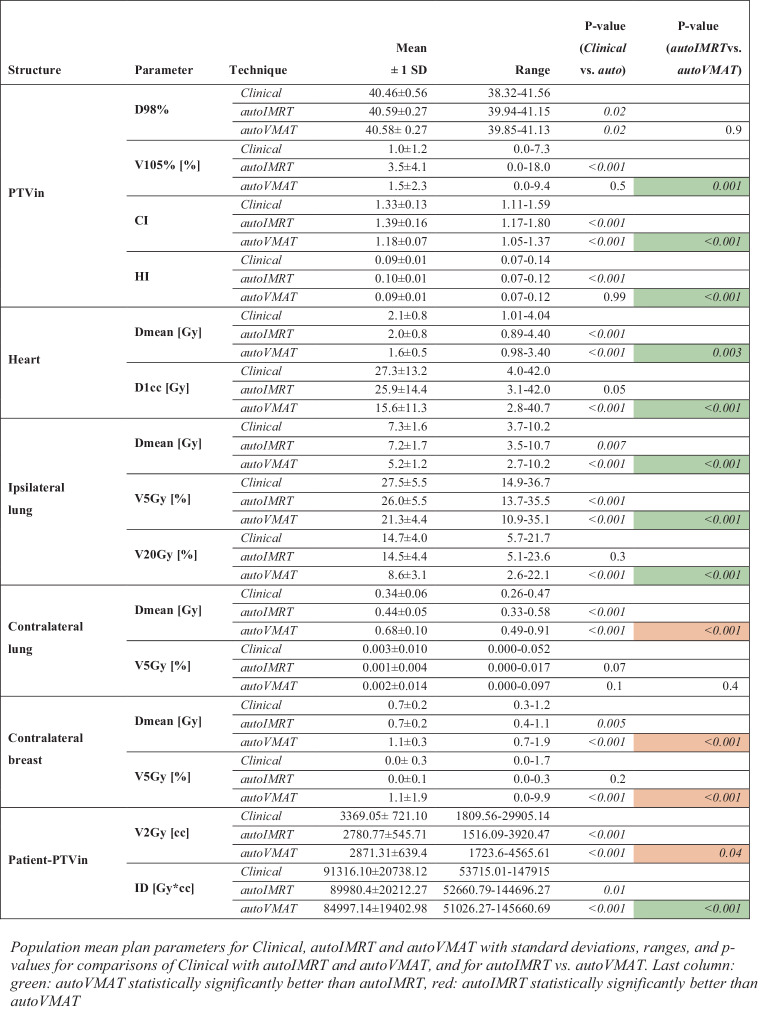
Population mean plan parameters for Clinical, autoIMRT, and autoVMAT with standard deviations, ranges, and *p*-values for comparisons of Clinical with autoIMRT and autoVMAT, and for autoIMRT vs. autoVMAT. *Last column* *green*: autoVMAT statistically significantly better than autoIMRT, *red*: autoIMRT statistically significantly better than autoVMAT. *autoIMRT* automated intensity modulated radiotherapy, *autoVMAT* automated volumetric modulated arc therapy, *PTVin* generated with 5 mm margin to the CTV and then cropped 5 mm from the skin surface, *Dx%* dose covering *x*% of the volume, *Vx%* volume receiving *x*% of prescription dose, *CI* ratio between the 95% isodose volume and PTV volume, *HI* difference between D2% and D98% divided by the prescription dose, *D1cc* the minimum dose to 1 cc, *VxGy* volume receving *xGy*, *ID* mean dose times volume

Dosimetric plan parameters for the clinical, autoIMRT, and autoVMAT plans show that the autoplans were similar or higher in quality than the clinical plans (Fig. 1). This observation demonstrates that the comparisons in this study between IMRT and VMAT for WBI with DIBH were based on treatment plans of sufficiently high dosimetric quality.

### autoIMRT vs. autoVMAT—dosimetric plan parameters

**Fig. 2:**
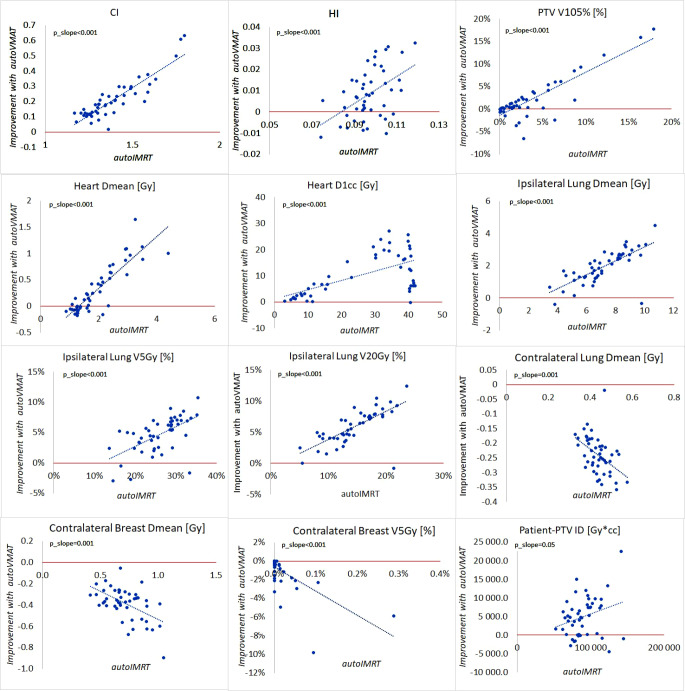
Comparisons between autoVMAT and autoIMRT for plan parameters with statistically significance differences. In each panel, each patient is represented by a marker. Along the x‑axes, parameter values for autoIMRT are depicted, while the y‑axes show improvements for treatment with autoVMAT instead of autoIMRT. The *straight dotted lines* in the plots were derived with linear regression analysis. Statistical significance for non-zero slopes is indicated by *p*_slope values. *autoIMRT* automated intensity modulated radiotherapy, *autoVMAT* automated volumetric modulated arc therapy, *PTVin* generated with 5 mm margin to the CTV and then cropped 5 mm from the skin surface, V*x%* volume receiving *x*% of prescription dose, *CI* ratio between the 95% isodose volume and PTV volume, *HI* difference between D2% and D98% divided by the prescription dose, *D1cc* the minimum dose to 1 cc, *VxGy* volume receving *x*Gy, *ID* mean dose times volume

AutoVMAT was clearly superior for the PTVin, heart, and ipsilateral lung (Fig. 1 and 2). While the techniques had on averege similar near-minimum PTVin doses (D98%), V105% and the CI went down from 3.5% to 1.5% and from 1.39 to 1.18, respectively. The heart Dmean and near-maximum dose, D1cc, reduced from 2.0 Gy to 1.6 Gy and from 25.9 Gy to 15.6 Gy, respectively. For the ipsilateral lung, observed Dmean, V5Gy, and V20Gy for autoIMRT/autoVMAT were 7.2/5.2 Gy, 26.0/21.3%, and 14.5/8.6%, respectively. These improvements with autoVMAT were accompanied by some deteriorations for the contralateral lung (Dmean: 0.44/0.68 Gy) and breast (Dmean: 0.7/1.1 Gy, V5Gy: 0.0/1.1%). Patient-PTVin ID went down from 89980 Gy*cc for autoIMRT to 84997 Gy*cc for autoVMAT, while the increase in V2Gy was not statistically significant.

**Fig. 3:**
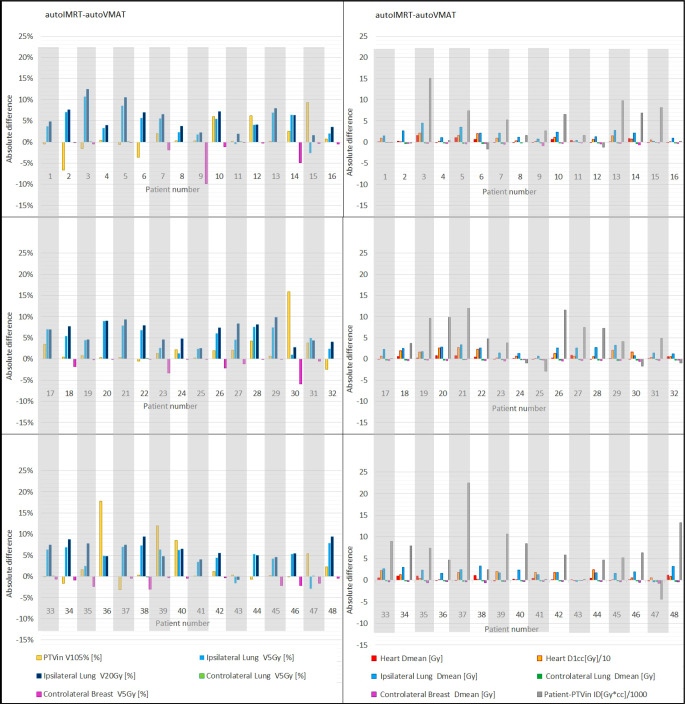
Differences in dosimetric plan parameters between autoIMRT and autoVMAT plans for each of the 48 study patients. Positive values are in favor of autoVMAT. Note: presented heart D1cc values were divided by 10 and patient-PTVin ID values were divided by 1000. *autoIMRT* automated intensity modulated radiotherapy, *autoVMAT* automated volumetric modulated arc therapy, *PTVin* generated with 5 mm margin to the CTV and then cropped 5 mm from the skin surface, *Vx%* volume receiving *x*% of prescription dose, *D1cc* the minimum dose to 1 cc, *VxGy* volume receving *xGy*, *ID* mean dose times volume

**Fig. 4:**
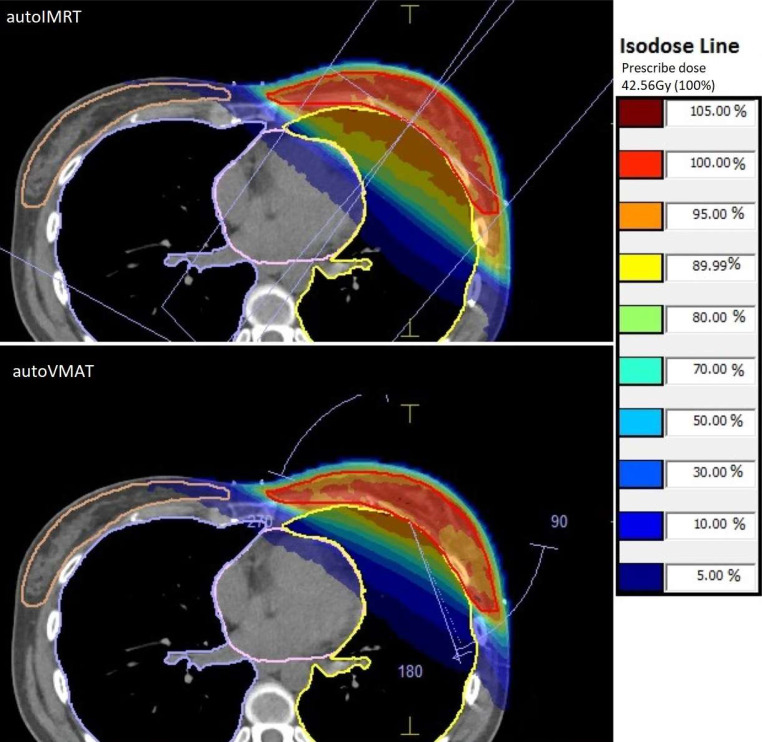
Axial dose distributions for autoIMRT and autoVMAT plans for the patient with the largest reduction in mean dose in ipsilateral lung when treating with autoVMAT instead of autoIMRT (reduction from 10.7 Gy to 6.2 Gy). *autoIMRT* automated intensity modulated radiotherapy with two tangential fields, *autoVMAT* automated volumetric modulated arc therapy with two small tangential subarc

Fig. 2 shows that improved dose delivery in PTVin, heart, and ipsilateral lung with autoVMAT was observed for almost all patients. The maximum absolute reduction with autoVMAT in hotspot volume (PTVin V105%) was 18%. With autoVMAT, CI improved for all patients, going from 1.80 to 1.17 for the patient benefitting most from autoVMAT. HI also improved for most patients, with an average deterioration of 0.006 for 12 patients. The maximum improvement in heart Dmean with autoVMAT was a reduction from 3.27 Gy to 1.62 Gy; 17 patients showed increases below 0.17 Gy. All patients improved in the near-maximum heart dose (D1cc), with a maximum reduction of 27 Gy. For a group of 13 patients with a heart D1cc between 30 and 40 Gy for treatment with autoIMRT, reductions with autoVMAT were in the range of 7–24 Gy (Fig. 2). For PTVin V105%, CI, HI, and all parameters for the heart and ipsilateral lung, dosimetric gains with autoVMAT were overall largest for patients with highest autoIMRT values (positive slopes Fig. 2). For most patients, dosimetric differences between autoIMRT and autoVMAT in the contralateral structures and the patient-PTVin were minor (Fig. 2). The observed absolute differences in dosimetric plan parameters for each of the study patients separately are shown in Fig. 3, demonstrating for most patients relatively large gains for autoVMAT in PTVin hotspots, heart, and ipsilateral lung at the price of minor losses in contralateral breast and lung. Fig. 4 shows dose distributions for one of the patients, illustrating enhanced sparing of the ipsilateral lung when using autoVMAT.

### autoIMRT vs. autoVMAT—risks for radiation-induced side effects

Compared to autoIMRT, mean heart doses and mean ipsilateral lung doses in autoVMAT plans were on average reduced by 0.4 Gy (range: −0.2–1.6 Gy) and 2.0 Gy (range: −0.4–4.5 Gy), respectively. Based on the published dependencies of ERR on dose described in the Materials and methods section, autoVMAT then resulted in estimated average ERR reductions for major coronary events and ipsilateral lung tumors of 3% (range: −1–12%) and 17% (range: −3–38%), respectively.

### autoIMRT vs. autoVMAT—plan deliverability, monitor units, and dose delivery times

There were no significant differences between autoIMRT and autoVMAT in γ passing rates and mean γ (Table 1). All autoIMRT plans passed the primary pretreatment QA test criteria, and only one autoVMAT plan failed. However, the latter plan passed the QA test at gantry zero to avoid angular dependency of the detector array (see Materials and methods section). AutoVMAT required more than twice the MU and a mean increase of 16 s per beam in delivery time compared to autoIMRT.

**Table 1:** Dosimetric verification results (γ analyses), MU, and beam delivery times for autoIMRT and autoVMAT plans of 10 patients. Applied definition of MU: 1 cGy/MU at isocenter with SSD = 90 cm and depth 10 cm

Parameter	Technique	Mean ± 1 SD	Range	*P*-value (autoIMRT vs. autoVMAT)
γ passing rate (%)	autoIMRT	98.6 ± 2.2	92.6–100	–
	autoVMAT	97.7 ± 4.1	86.3–100	0.6
Mean γ	autoIMRT	0.33 ± 0.06	0.24–0.48	–
	autoVMAT	0.32 ± 0.08	0.24–0.51	0.2
MU	autoIMRT	273 ± 10	260–290	–
	autoVMAT	675 ± 57	563–734	*0.007*
Beam delivery time (s)	autoIMRT	52 ± 10	33–73	–
	autoVMAT	68 ± 8	52–86	*<* *0.001*

*italicized p-values* statistical significance of plan parameter differences (Wilcoxon’s signed-rank tests, *p* < 0.05)

*autoIMRT* automated intensity modulated radiotherapy with two tangential fields, *autoVMAT* automated volumetric modulated arc therapy with two small tangential subarc

## Discussion

In this study, we compared 48 left-sided breast cancer patients treated with DIBH for WBI with two tangential IMRT fields or with two small tangential VMAT arcs, using high-quality treatment plans that were automatically generated with a multi-criterial optimizer. By using the same optimization scheme (wish-list) for both treatment approaches, bias towards higher plan quality for one of the approaches could be minimized. Applied autoIMRT and autoVMAT plans had similar or higher quality than the clinical plans, in line with previous studies that showed significant quality improvements compared to manual planning [35–40].

Compared to autoIMRT, autoVMAT demonstrated significant improvements in dose homogeneity in the ipsilateral breast, which could potentially result in reduced toxicity, as previously observed in randomized clinical trials [7–10]. CI was also improved with autoVMAT, which is in line with the observed enhanced sparing of heart and ipsilateral lung. We estimated that the reductions in ipsilateral lung Dmean obtained with autoVMAT would on average result in an ERR reduction for developing an ipsilateral lung tumor of 16% (33% for smokers [12]), with reductions up to 38% for individual patients. The reductions in mean heart doses were estimated to result in an average ERR decrease for major coronary events of 3% (maximum 12%). According to QUANTEC guidelines, a heart V25Gy below 10% can result in cardiac mortality below 1% in long-term follow-up after RT [43]. Interestingly, regression analyses demonstrated that dosimetric improvements with autoVMAT in ipsilateral breast, ipsilateral lung, and heart were largest for patients with the highest doses in the autoIMRT plans (Fig. 2). Apparently, patients who suffered most from the use of fixed beam angles in IMRT benefitted most from the enhanced rotational freedom with VMAT. Some deterioration for the contralateral breast dose was observed for autoVMAT (increase of Dmean by 0.4 Gy). Because of the lack of data obtained from large patient cohorts, we were not able to provide robust estimates of enhanced risks of secondary contralateral breast cancer induction due to this dose increase. However, we believe that for most patients, the observed dosimetric advantages of autoVMAT in other structures outweigh the generally small dose increase in contralateral breast. For patient-PTVin, Dmean slightly improved with autoVMAT and V2Gy slightly deteriorated. The significant increase in delivered MU with autoVMAT results in higher low doses. As these doses are low anyway, the impact of the increased leakage may be considered minor compared to the dose decreases in lung and heart as reported above. The small number of MU for autoIMRT plans compared to autoVMAT plans is probably due to the choice of using step-and-shoot IMRT, since when the delivery is continuous (as for VMAT or dynamic IMRT), the number of MU significantly increases.

The observed increase in delivery time for autoVMAT compared to autoIMRT was on average 16 s per beam. In DIBH treatments, the required number of breath-holds for delivery of the total fraction dose is an important factor. The time that patients can hold their breath can largely vary from patient to patient. When assuming that an average breath-hold time is 30 s, seven measured autoIMRT and only one autoVMAT plan could be treated with two breath-holds per beam, while all the other treatments would require at least three breath-holds per beam.

Although the data suggest an overall dosimetric advantage for VMAT over IMRT, there are some patients that were maybe better off with IMRT (Fig. 3). Automated treatment planning could be applied for personalization of treatment technique without an increase in planning workload. For this purpose, autoVMAT and autoIMRT plans could be automatically generated for each patient, followed by a posteriori selection of the best, patient-specific approach.

Of the five published planning studies [15–19] comparing tangential IMRT with tangential VMAT for left-sided WBI, only Yu et al. [18] and Vikström et al. [17] investigated DIBH treatments, current start-of-the-art practice for left-sided WBI. The differences between IMRT and VMAT plans, as observed by Yu et al. [18] in 14 patients, are mostly rather different from those in our study: no differences in PTVin homogeneity, dose conformity, heart dose, and contralateral breast and lung Dmean, while these parameters were clearly favorable in our autoVMAT plans, except for contralateral structures. As also observed in our study, they found enhanced contralateral breast V5Gy, but both the observed V5Gy values and the differences were very different from our study: 0.0/1.1% for autoIMRT/autoVMAT in our study vs. 2.2/9.1% for IMRT/VMAT by Yu et al. These differences may be related to differences in gantry angles favoring lower contralateral breast dose in our study. They found that VMAT was favorable for ipsilateral lung V30Gy, which aligns with our observations. In contrast to our study, the total MU was significantly higher with IMRT, which could possibly be influenced by the use of sliding-window IMRT by Yu et al., while step-and-shoot IMRT was used in our study. In a study based on 16 cases, Vikström et al. [17] observed no advantages for VMAT except for a small improvement in target coverage (V95%), again very different from the advantages of VMAT seen in our study. A limitation of the aforementioned studies is that the results are based on relatively small patient groups (14–16 patients), whereas our study investigated a larger group of 48 patients.

In none of the five published planning studies comparing tangential IMRT with tangential VMAT for left WBI [15–19] were the treatment plans automatically generated, leaving room for bias and inconsistent plan quality. Another large difference was the number of patients included in our study. In all five studies, the total prescribed dose was 50.0 Gy, delivered in 2.0 Gy daily fractions, which is different from currently widely applied hypofractionated regimes such as 42.56 Gy in 16 daily fractions (used in this study) or 40 Gy in 15 daily fractions. In none of the comparisons were corresponding IMRT and VMAT dose distributions normalized for equal PTVin coverage, which may have had an important impact on reported differences in OAR doses.

The angles of the tangential autoIMRT beams were the same as clinically selected by the planner, and therefore manually optimized for each patient. Although it was previously demonstrated that clinical angles were mostly adequate [27], an automatic beam angle optimization approach could possibly have resulted in improved autoIMRT plans. With autoVMAT there is no need for patient-specific beam angle selection, which is a significant practical advantage. The clinical isocenter was used for both autoIMRT and autoVMAT, limiting bias in the comparisons. On the other hand, an automated procedure for isocenter selection could have resulted in better overall plans [24, 25].

A further extension of the current study could be including contouring of heart substructures that is spreading in clinical practice. Some guidelines recommend to also take into account constraints for cardiac subvolumes, e.g., the left anterior descending artery (LAD) in breast cancer radiotherapy [44]. Unfortunately, substructures of the heart were not delineated for the patients in this study. Precisely contouring some of them (for example LAD) is challenging in daily practice and in clinical research, since they are hardly discernible on non-contrast simulation CT scans, and manual delineation is often imprecise, poorly reproducible, and time consuming [45].

The observed advantages of VMAT over IMRT were obtained for patients treated with DIBH and daily image guidance using both CBCT and an optical monitoring system, allowing a PTV margin of 0.5 cm. The planning aims and applied plan evaluation parameters were in line with current global practice. It is not clear to what extent plan quality would be influenced by choosing (slightly) different planning and image-guidance approaches. We believe that the promising results of this study could stimulate further research to find definitive answers to the IMRT/VMAT question.

It is well known that modulated plans can suffer from limited robustness against respiratory motion, anatomical changes such breast swelling, uncertainties in patient positioning, and limited breath-hold reproducibility. Several papers deal with the analysis of IMRT and VMAT plan robustness in breast radiotherapy, either simulating patient displacements or deformations or recalculating plans on CBCTs [46–48].

Kügele M et al. observed a good reproducibility of the intra-fractional DIBH isocenter using an optical system to follow the movement of the external surface during delivery in DIBH and set tolerance levels on the isocenter displacements [46]. According to Van der Veen et al., VMAT with proper handling of the skin flash is as robust as tangential IMRT [47]. In the work by Rossi et al., the dosimetric effect of soft tissue deformation and breast tissue swelling was similar for VMAT and field-in-field plans if 3D image matching was used [48]. Routine use of daily CBCT reduces dosimetric effects of daily variations, suggesting safe use of modulated techniques in breast radiotherapy. Intrafractional monitoring of patient surface position using optical systems can help to further reduce the dosimetric effect. Both daily CBCT and an optical system are routinely used in our clinical practice for WBI with DIBH.

In this study, we have used flash margins, daily CBCT, and DIBH with optical steering and verification to enhance treatment delivery accuracy and robustness. At this point we believe that these measures would allow safe and effective delivery of both autoVMAT and autoIMRT plans. However, this needs verification, which is being performed in on-going comprehensive robustness analysis, considering that for IMRT the build-up zone moves together with the breast without affecting PTVin coverage, which could potentially be different for VMAT. Results will be presented in a future publication.

## References

[1] Naghavi M, Abajobir AA, Abbafati C (2017). Global, regional, and national age-sex specifc mortality for 264 causes of death, 1980–2016: a systematic analysis for the Global Burden of Disease Study 2016. Lancet.

[2] Fisher B, Anderson S, Bryant J (2002). Twenty-year follow-up of a randomized trial comparing total mastectomy, lumpectomy, and lumpectomy plus irradiation for the treatment of invasive breast cancer. N Engl J Med.

[3] Mayo CS, Urie MM, Fitzgerald TJ (2005). Hybrid IMRT plans—concurrently treating conventional and IMRT beams for improved breast irradiation and reduced planning time. Int J Radiat Oncol Biol Phys.

[4] Kestin LL, Sharpe MB, Frazier RC (2000). Intensity modulation to improve dose uniformity with tangential breast radiotherapy: initial clinical experience. Int J Radiat Oncol Biol Phys.

[5] Dayes I, Rumble RB, Bowen J (2012). Intensity-modulated radiotherapy in the treatment of breast cancer. Clin Oncol.

[6] Fogliata A, Seppälä J, Reggiori G (2017). Dosimetric trade-offs in breast treatment with VMAT technique. Br J Radiol.

[7] Pignol JP, Olivotto I, Rakovitch E (2008). A multicenter randomized trial of breast intensity-modulated radiation therapy to reduce acute radiation dermatitis. J Clin Oncol.

[8] Donovan E, Bleakley N, Denholm E (2007). Randomised trial of standard 2D radiotherapy (RT) versus intensity modulated radiotherapy (IMRT) in patients prescribed breast radiotherapy. Radiother Oncol.

[9] Harsolia A, Kestin L, Grills I (2007). Intensity-modulated radiotherapy results in significant decrease in clinical toxicities compared with conventional wedge-based breast radiotherapy. Int J Radiat Oncol Biol Phys.

[10] Mukesh MB, Barnett GC, Wilkinson JS (2013). Randomized controlled trial of intensity-modulated radiotherapy for early breast cancer: 5-year results confirm superior overall cosmesis. J Clin Oncol.

[11] Darby S, McGale P, Correa C (2011). Effect of radiotherapy after breast-conserving surgery on 10-year recurrence and 15-year breast cancer death: meta-analysis of individual patient data for 10 801 women in 17 randomised trials. Lancet.

[12] Grantzau T, Thomsen MS, Væth M, Overgaard J (2014). Risk of second primary lung cancer in women after radiotherapy for breast cancer. Radiother Oncol.

[13] Mast ME, Van Kempen-Harteveld L, Heijenbrok MW (2013). Left-sided breast cancer radiotherapy with and without breath-hold: Does IMRT reduce the cardiac dose even further?. Radiother Oncol.

[14] Zagar T, Tang XI, Jones E (2015). Prospective assessment of deep inspiration breath hold to prevent radiation-associated cardiac perfusion defects in patients with left-sided breast cancer. J Clin Oncol.

[15] Virén T, Heikkilä J, Myllyoja K (2015). Tangential volumetric modulated arc therapy technique for left-sided breast cancer radiotherapy. Radiat Oncol.

[16] Zhao H, He M, Cheng G (2015). A comparative dosimetric study of left sided breast cancer after breast-conserving surgery treated with VMAT and IMRT. Radiat Oncol.

[17] Vikström J, Hjelstuen MH, Wasbø E (2018). A comparison of conventional and dynamic radiotherapy planning techniques for early-stage breast cancer utilizing deep inspiration breath-hold. Acta Oncol.

[18] Yu PC, Wu CJ, Tsai YL (2018). Dosimetric analysis of tangent-based volumetric modulated arc therapy with deep inspiration breath-hold technique for left breast cancer patients. Radiat Oncol.

[19] Supakalin N, Pesee M, Thamronganantasakul K (2018). Comparision of different radiotherapy planning techniques for breast cancer after breast conserving surgery. Asian Pac J Cancer Prev.

[20] Hussein M, Heijmen BJM, Verellen D, Nisbet A (2018). Automation in intensity modulated radiotherapy treatment planning—a review of recent innovations. Br J Radiol.

[21] Sharfo AWM, Dirkx MLP, Breedveld S (2017). VMAT plus a few computer-optimized non-coplanar IMRT beams (VMAT+) tested for liver SBRT. Radiother Oncol.

[22] Rossi L, Sharfo AW, Aluwini S (2018). First fully automated planning solution for robotic radiosurgery–comparison with automatically planned volumetric arc therapy for prostate cancer. Acta Oncol.

[23] Sharfo AWM, Dirkx MLP, Bijman RG (2018). Late toxicity in the randomized multicenter HYPRO trial for prostate cancer analyzed with automated treatment planning. Radiother Oncol.

[24] Purdie TG, Dinniwell RE, Letourneau D (2011). Automated planning of tangential breast intensity-modulated radiotherapy using heuristic optimization. Int J Radiat Oncol Biol Phys.

[25] Purdie TG, Dinniwell RE, Fyles A, Sharpe MB (2014). Automation and intensity modulated radiation therapy for individualized high-quality tangent breast treatment plans. Int J Radiat Oncol Biol Phys.

[26] Fogliata A, Nicolini G, Bourgier C (2015). Performance of a knowledge-based model for optimization of volumetric modulated arc therapy plans for single and bilateral breast irradiation. PLoS ONE.

[27] Penninkhof J, Spadola S, Breedveld S (2017). Individualized selection of beam angles and treatment isocenter in tangential breast intensity modulated radiation therapy. Int J Radiat Oncol Biol Phys.

[28] Marrazzo L, Meattini I, Arilli C (2019). Auto-planning for VMAT accelerated partial breast irradiation. Radiother Oncol.

[29] van Duren-Koopman MJ, Tol JP, Dahele M (2018). Personalized automated treatment planning for breast plus locoregional lymph nodes using hybrid RapidArc. Pract Radiat Oncol.

[30] Kisling K, Zhang L, Shaitelman SF (2019). Automated treatment planning of postmastectomy radiotherapy. Med Phys.

[31] Mizuno N, Yamauchi R, Kawamori J (2019). Evaluation of a new commercial automated planning software for tangential breast intensity-modulated radiation therapy. Radiol Phys Technol.

[32] Guo B, Shah C, Xia P (2019). Automated planning of whole breast irradiation using hybrid IMRT improves efficiency and quality. J Appl Clin Med Phys.

[33] Lin TC, Lin CY, Li KC (2020). Automated hypofractionated IMRT treatment planning for early-stage breast cancer. Radiat Oncol.

[34] Breedveld S, Storchi PRM, Voet PWJ, Heijmen BJM (2012). ICycle: integrated, multicriterial beam angle, and profile optimization for generation of coplanar and noncoplanar IMRT plans. Med Phys.

[35] Heijmen B, Voet P, Fransen D (2018). Fully automated, multi-criterial planning for volumetric modulated arc therapy—an international multi-center validation for prostate cancer. Radiother Oncol.

[36] Voet PWJ, Dirkx MLP, Breedveld S (2013). Toward fully automated multicriterial plan generation: A prospective clinical study. Int J Radiat Oncol Biol Phys.

[37] Buschmann M, Sharfo AWM, Penninkhof J (2018). Automated volumetric modulated arc therapy planning for whole pelvic prostate radiotherapy. Strahlenther Onkol.

[38] Sharfo AWM, Breedveld S, Voet PWJ (2016). Validation of fully automated VMAT plan generation for library-based plan-of-the-day cervical cancer radiotherapy. PLoS ONE.

[39] Della Gala G, Dirkx MLP, Hoekstra N (2017). Fully automated VMAT treatment planning for advanced-stage NSCLC patients. Strahlenther Onkol.

[40] Buergy D, Sharfo AWM, Heijmen BJM (2017). Fully automated treatment planning of spinal metastases—a comparison to manual planning of volumetric modulated arc therapy for conventionally fractionated irradiation. Radiat Oncol.

[41] Darby SC, Ewertz M, McGale P (2013). Risk of ischemic heart disease in women after radiotherapy for breast cancer. N Engl J Med.

[42] Taylor C, Duane FK, Dodwell D (2017). Estimating the risks of breast cancer radiotherapy: evidence from modern radiation doses to the lungs and heart and from previous randomized trials. J Clin Oncol.

[43] Gagliardi G, Constine LS, Moiseenko V (2010). Radiation dose-volume effects in the heart. Int J Radiat Oncol Biol Phys.

[44] Piroth MD, Baumann R, Budach W (2019). Heart toxicity from breast cancer radiotherapy: current findings, assessment, and preventio. Strahlenther Onkol.

[45] Lorenzen EL, Taylor CW, Maraldo M (2013). Inter-observer variation in delineation of the heart and left anterior descending coronary artery in radiotherapy for breast cancer: a multi-centre study from Denmark and the UK. Radiother Oncol.

[46] Kügele M, Edvardsson A, Berg L (2018). Dosimetric effects of intrafractional isocenter variation during deep inspiration breath-hold for breast cancer patients using surface-guided radiotherapy. J Appl Clin Med Phys.

[47] van der Veen GJ, Janssen T, Duijn A (2019). A robust volumetric arc therapy planning approach for breast cancer involving the axillary nodes. Med Dosim.

[48] Rossi M, Boman E, Skyttä T (2018). Dosimetric effects of anatomical deformations and positioning errors in VMAT breast radiotherapy. J Appl Clin Med Phys.

